# Long-term neurocognitive effects of methylphenidate in patients with attention deficit hyperactivity disorder, even at drug-free status

**DOI:** 10.1186/1471-244X-12-194

**Published:** 2012-11-09

**Authors:** Yu-Shu Huang, Liang-Jen Wang, Chih-Ken Chen

**Affiliations:** 1 Department of Child Psychiatry and Sleep Center, Chang Gung Memorial Hospital at Linko, Taoyuan, Taiwan; 2 Chang Gung University School of Medicine, Taoyuan, Taiwan; 3 Department of Psychiatry, Chang Gung Memorial Hospital at Keelung, Keelung, Taiwan; 4 Department of Psychiatry, Chang Gung Memorial Hospital at Keelung, No.200 Lane 208, Ji-Jin 1st Road, Anle District, Keelung City, 204, Taiwan, R.O.C

**Keywords:** ADHD, Cognition, Methylphenidate, Neuropsychological test, Subtypes, Predictor

## Abstract

**Background:**

Methylphenidate (MPH), a psycho-stimulant, is the most widely administered drug for the pharmacological management of patients with attention deficit hyperactivity disorder (ADHD). This study attempts to determine whether sustainable improvements occur in neurocognitive function among ADHD patients following 12-month treatment with MPH, at drug-free status. Whether age groups, gender or ADHD subtypes differ in neurocognitive performance during MPH treatment is also examined.

**Methods:**

Study participants consisted of 103 ADHD patients (mean age: 9.1 ± 1.9 years old) who were drug naïve or drug free for at least 6 months. The patients were prescribed oral short-acting MPH at each dose range of 0.3–1.0 mg/kg daily. During 12 months of the study, the patients underwent the test of variables of attention (TOVA) at the baseline, month 6 and month12. Patients were instructed to not intake MPH for one week before the second and the third TOVA.

**Results:**

Seventy five patients completed the study. Results of this study indicated that although commission errors and response sensitivity (d’) significantly improved during MPH treatment for 12 months, omission errors, response time, response time variability and ADHD score did not. While younger ADHD patients (<9 y/o) performed better in response time, response time variability, d’ and ADHD score than older ones (≥9 y/o), the latter more significantly improved in response time than the former during 12 months of treatment. Additionally, boys improved more than girls in omission error and d’. Moreover, although ADHD subtypes significantly differed in ADHD score during the treatment, MPH treatment and ADHD subtypes did not interact with each other for all TOVA indices.

**Conclusions:**

ADHD patients significantly improved in impulsivity and perceptual sensitivity, determined as TOVA, during MPH treatment for 12 months. Age and gender, yet not ADHD subtypes, appear to influence the MPH treatment effects in some indices of TOVA. A future study containing a comparison group is suggested to confirm whether the neurocognitive improvements are attributed to long-term effects of MPH or natural maturation of patients.

## Background

Attention deficit hyperactivity disorder (ADHD) is a common neuropsychiatric disorder among children and adolescents, affecting 3% to 10% of all school-age children [1]. Numerous neuropsychological tests suggest that children inflicted with ADHD have significant cognitive impairments [2]. The current Diagnostic and Statistical Manual, Fourth Edition (DSM-IV) [3] classifies ADHD into 3 subtypes: inattentive type, hyperactive-impulsive type and combined type, according to the predominant clinical manifestations of inattention, hyperactivity, and impulsivity. Despite the use of neuropsychological methods to identify differentiating endophenotypes among ADHD patients [4], whether there are distinct neuropsychological deficits between ADHD subtypes is a contentious issue [5-7].

Methylphenidate (MPH), a psycho-stimulant, is the most widely administered drug for the pharmacological management of ADHD patients [8]. The pharmacological profile acts in dual mechanisms by inhibiting the re-uptake of dopamine and norepinephrine [9]. MPH exerts treatment effects for both behavioral and cognitive dimensions in ADHD patients [10,11]. Numerous studies indicate that various domains of neurocognitive function are enhanced under acute MPH challenge in ADHD patients [12-15]. As is generally assumed, behavioral symptoms or neurocognitive impairment of ADHD patients returns immediately when the drug effect diminishes. However, some studies have investigated the neurocognitive effects over long-term MPH treatment, with a lack of consensus in those findings. The pilot study of Aggarwal and Lillystone indicated that the commission errors (yet not omission errors, response time or variability) significantly ameliorated after stimulant medication therapy at least for 12 months [16]. Konrad *et al.* demonstrated that children with ADHD did not substantially improve in executive control performance after long-term MPH treatment [17]. However, that study was limited by a small sample size. Moreover, Zhang *et al.* noted that intelligence quotient of ADHD patients significantly increased after 6 months of MPH treatment [18]. In sum, whether long-term prescription of MPH enhances neurocognitive performance in ADHD patients remains unclear.

The treatment effects are diverse among ADHD patients, with some studies attempting to determine the predictors of treatment outcome [19,20]. Exactly how MPH affects the neuropsychological profiles between subtypes of ADHD patients still remains a contentious issue. Several studies demonstrated that MPH enhanced cognitive performance equally in patients with ADHD-combined type and ADHD-inattentive type [21-23]. However, other studies demonstrated that patients with ADHD-combined type significantly improved in behavioral symptoms [24] and executive function performance [25]. Furthermore, although some cross-sectional studies indicated that age and gender were associated with the neurocognitive functions in ADHD patients [26,27], whether these factors influence the long-term treatment effects of MPH remains unclear.

Therefore, this study attempts to determine whether neurocognitive function among ADHD patients improve in a sustainable manner during 12 months of treatment with MPH, at drug-free status. In addition to the main purpose, we also conduct an explorative analysis to examine whether age groups, gender or ADHD subtypes differ in neurocognitive performance during MPH treatment.

## Methods

### Study participants

ADHD patients between ages of 6 and 16 years old were recruited from the out-patient Child Psychiatry Department at Chang Gung Children’s Hospital (Linkou, Taiwan). The study received approval from the Institutional Review Board of Chang Gung Memorial Hospital. Written informed consent was obtained from the parents of the patients. ADHD and comorbid disorders were diagnosed by two senior child psychiatrists, based on the DSM-IV criteria [3] after structured interviews with the Chinese version of the Schedule for Affective Disorder and Schizophrenia for School-Age Children, epidemiologic version (K-SADS-E) [28]. The Chinese version of K-SADS-E was developed by the Child Psychiatry Research Group in Taiwan [29]. ADHD subjects were classified as inattentive, hyperactive-impulsive, or combined type. Patients were excluded from the study if they had a history of co-morbid pervasive developmental disorders or mental retardation, as well as those who had a history of bipolar disorder, psychosis, epilepsy, or brain injury. Included patients were either newly diagnosed with ADHD or had an existing diagnosis, yet had not taken ADHD medication during the previous 6 months or longer.

### Measurements

Neurocognitive function of ADHD patients was assessed using the test of variables of attention (TOVA)-Visual [30], which is a computerized, continuous performance test comprising a target stimulus and a non-target stimulus. Experienced child psychologists conducted TOVA with individual subjects in a room dedicated to reducing variability in testing conditions.

The TOVA stimuli are coloured squares with a small black square within, which is adjacent to either the top or the bottom edge. The squares with a small inner square near the top edge are designated targets, and the ones with the small squares near the bottom edge are non-targets. The stimuli appear individually and are presented randomly, based on a determined ratio. The tested subject was instructed to immediately press a button after seeing a target and does not respond when a non-target is presented. The test lasted 22.6 minutes and is preceded by a practice session for 2.5 minutes. Two visual stimuli appeared 648 times. The TOVA was reported to achieve satisfactory levels of reliability and concurrent validity in Taiwanese children with ADHD [31]. The indices measured in the TOVA include the following:

***Omission errors***: this score is evaluated as the failure to respond to the target stimulus. Omission error scores are presented as percentages and are considered to be a measure of inattention.

***Commission errors***: this score is measured as an inappropriate response to the non-target stimulus. Commission error scores are presented as percentages and are considered to reflect impulsivity or disinhibition.

***Response time*** (in msec): this score is determined as the average of the correct response times. This score denotes response latency in information processing and motor response speed.

***Response time variability***: this score is evaluated as the standard deviation of the mean of correct response times. It is a measure of the subject’s inconsistency in response times.

***Response sensitivity (d’)***: this score is a response sensitivity score reflecting the ratio of the hit rate to false alarm rate. This score refers to the accuracy of target and non-target discrimination and is interpreted as a measure of perceptual sensitivity.

***ADHD score***: this score is a composite score generated by the TOVA program. The score is calculated by comparing an individual’s performance on the TOVA to those of an ADHD sample collected by the authors of the TOVA. The score describes how similar an individual’s performance is to the ADHD profile.

### Study procedure

Each subject performed the TOVA 3 times. At the first TOVA (month 0), all subjects were drug naïve or had not taken medication for ADHD during the previous 6 months or longer. Subsequently, ADHD patients were prescribed oral short-acting methylphenidate (MPH) twice or three times daily at each dose range of 0.3–1.0 mg/kg, based on the severity of their clinical symptoms as well as their age, height, and body weight. Concomitant medications were prohibited. Patient care was performed based on their usual practice at the out-patient services of the child psychiatry department. There was no additional behavioral therapy or family therapy provided during the period of study. Drug compliance at each visit was confirmed according to the reports of patients’ caregivers.

The second and third TOVA were performed at the 6^th^ and 12^th^ month after treatment with MPH, respectively. Patients were instructed to not intake MPH for one week before the second and the third TOVA. The TOVA tests were compared before treatment, 6 months and 12 months after MPH treatment.

### Statistical analysis

Data were analyzed using the statistical software package SPSS, version 16.0 (SPSS Inc., Chicago, IL, USA). Variables are presented as either the mean (standard deviation) or frequency. Two-tailed p values of <0.05 were considered statistically significant.

The TOVA results were reported as standard deviations, which indicated the extent of deviation from the norm. Variables between ADHD subtypes at baseline were compared using the Fisher’s Exact Test and Kruskal Wallis Test. Missing data at the 6^th^ and 12^th^ months were accounted for by using the method of last observation carried forward (LOCF). The longitudinal data were analyzed using a linear mixed model, with the maximum likelihood estimation method and auto-regression covariance matrix, as the primary analytic strategy. To examine the trends of neuro-cognitive function under MPH treatment within 12 months, the dependent variables were set as the indices in TOVA. The age of patients was divided into a categorical variable based on the median age of 9. This study also investigated the extent of the differences in changes of performance in TOVA between age groups, gender and ADHD subtypes, also by the linear mixed model. The hypothesis that a differential change occurs in dependent measures over 12 months is supported by significant interactions of MPH treatment × age, gender or ADHD subtypes.

## Results

This study recruited 103 ADHD patients (mean age: 9.1 ± 1.9 years). Of the 103 patients, 82 (79.6%) were male and 21 (20.4%) were female. Fifty-four of them were inattentive type; 5 were hyperactive-impulsive type; and 44 were combined type. The three ADHD subtypes identified at month 0 (pretreatment) did not significantly differ in age, gender, and indices in TOVA (Table 1). Owing to the small number of patients with hyperactive-impulsive type, these five patients were placed with patients with combined type into further longitudinal analysis.

**Table 1:** Age, gender and performance in TOVA for children with ADHD between subtypes at baseline

	**Inattentive type (n = 54)**	**Hyperactive-impulsive type (n = 5)**	**Combined type (n = 44)**	**Statistic**^**a**^
Age (years)	9.4 (1.9)	8.8 (1.5)	8.7 (2.0)	3.31 (0.192)
Gender (female/male), n (%)	14 (25.9)/40 (74.1)	0 (0)/5 (100)	7 (15.9)/37 (84.1)	2.19 (0.308)
Omission errors	−1.01 (1.52)	−1.89 (2.18)	−1.11 (1.52)	0.80 (0.672)
Commission errors	−0.06 (1.42)	−0.37 (1.41)	−0.41 (1.60)	4.68 (0.097)
Response time	−0.76 (1.25)	−0.43 (0.90)	−0.96 (1.61)	0.77 (0.682)
Response time variability	−0.87 (1.53)	−0.95 (0.76)	−1.31 (1.45)	1.90 (0.387)
Response sensitivity (d’)	−0.74 (0.93)	−1.02 (1.02)	−0.97 (0.81)	0.94 (0.626)
ADHD score	−1.49 (2.94)	−1.52 (2.27)	−3.32 (3.60)	3.77 (0.152)

Note: TOVA results are reported as standard deviations from the norm; data are expressed as mean (SD) or n (%); ^a^ statistical values are expressed as *χ*^2^ (p-value) using Kruskal Wallis Test or Fisher’s Exact Test.

Among the 103 ADHD patients at the initial visit, 75 patients remained in the study at month 6 and month 12. The mean dose of MPH of the 75 patients at the endpoint is 19.8 ± 13.2 mg. Among the 28 drop-out patients, 10 patients withdrawal of consent, and 18 patients lost of follow up. Comparing the remaining patients with the drop-out patients, there were no significant differences in demographic characteristics (age: t = 0.16, p = 0.877; gender: *χ*^2^ = 0.33, p = 0.758; ADHD subtypes: *χ*^2^ = 0.24, p = 0.807) and performances in TOVA (omission errors: t = 0.79, p = 0.434; commission errors: t = −0.37, p = 0.709; response time: t = −0.49, p = 0.624; response time variability: t = −1.95, p = 0.054; response sensitivity (d’) : t = −0.68, p = 0.496; ADHD score: t = −1.25, p = 0.215).

Of the indices in TOVA, commission errors (F = 3.66, p = 0.029) and d’ (F = 3.71, p = 0.026) were significantly improved during 12 months of treatment by MPH in a clinical setting. Table 2 summarizes the performance in TOVA at months 0, 6 and 12. Post-hoc tests indicated that d’ improved significantly (t = 2.71, p = 0.007) after 6 months of treatment with MPH. Compared with data at month 0, commission errors improved significantly (t = 2.70, p = 0.008) after 12 months of treatment with MPH. Additionally, omission errors, response time, response time variability and ADHD score at the endpoint did not significantly improve. Moreover, all indices of TOVA did not change significantly between months 6 and 12.

**Table 2:** TOVA performance for patients with ADHD at baseline, and at months 6 and month 12 after methylphenidate treatment

	**Month 0 (N = 103)**	**Month 6 (N = 75)**	**Month 12 (N = 75)**	**Statistic**^**a**^	**Post-hoc tests**
Omission errors	−1.09 (1.55)	−1.00 (1.47)	−1.17 (1.61)	0.90 (0.408)	NS
Commission errors	−0.22 (1.49)	0.01 (1.22)	0.16 (1.05)	3.66 (0.029)*	Month 12 > Month 0 **
Response time	−0.83 (1.40)	−0.75 (1.24)	−0.79 (1.23)	0.43 (0.654)	NS
Response time variability	−1.06 (1.47)	−0.91 (1.31)	−0.99 (1.36)	1.17 (0.315)	NS
Response sensitivity (d’)	−0.85 (0.88)	−0.65 (0.92)	−0.67 (0.93)	3.71 (0.026)*	Month 6 > Month 0 **
ADHD score	−2.22 (3.29)	−2.00 (3.08)	−2.09 (3.23)	0.95 (0.390)	NS

Note: TOVA results are reported as standard deviations from the norm, and expressed as mean (SD); month 0 data is pretreatment data; ^a^ statistical values are expressed as F (p-value) using linear mixed model analysis; NS = non-significant; *p<0.05, **p<0.01.

Table 3 summarizes the effects of age, gender, ADHD subtypes and MPH treatment on each index of TOVA. Younger ADHD patients (<9y/o) performed better in response time (t = 2.34, p = 0.020), response time variability (t = 2.51, p = 0.013), d’ (t = 2.57, p = 0.011), and ADHD score (t = 2.77, p = 0.006) than older patients (≥9 y/o). However, younger patients improved less in response time (t = −2.72, p = 0.007) than older ones during 12 months of treatment. In terms of gender, the gender groups did not significantly differ in any TOVA index. Nevertheless, male patients more significantly improved in omission error (t = 2.30, p = 0.022) and d’ (t = 2.41, p = 0.017) than female patients during treatment. As for the effects of ADHD subtypes, ADHD subtypes (t = 2.21, p = 0.029) significantly differed in ADHD score during 12 months of treatment with MPH. Moreover, ADHD subtypes did not significantly differ in other indices of TOVA. Furthermore, all TOVA indices revealed no effects of interaction between MPH treatment and ADHD subtypes.

**Table 3:** The effects of age groups, gender, ADHD subtypes and methylphenidate (MPH) treatment on each index of TOVA

	**Omission errors**	**Commission errors**	**Response time**	**Response time variability**	**Response sensitivity (d’)**	**ADHD score**
Age (<9 years vs. ≥9 years)	−0.30 (0.764)	0.51 (0.610)	2.34 (0.020)*	2.51 (0.013)*	2.57 (0.011)*	2.77 (0.006)**
Gender (female vs. male)	−0.18 (0.858)	0.91 (0.365)	0.44 (0.658)	0.96 (0.336)	0.50 (0.621)	−0.10 (0.924)
ADHD subtypes ^a^	0.17 (0.862)	1.19 (0.234)	0.79 (0.432)	1.50 (0.136)	1.62 (0.107)	2.21 (0.029)*
MPH treatment	−0.14 (0.890)	2.02 (0.044)*	1.57 (0.119)	1.16 (0.247)	2.24 (0.026)*	1.42 (0.158)
Age × MPH treatment	−0.16 (0.870)	0.58 (0.561)	−2.72 (0.007)**	−1.24 (0.218)	−0.76 (0.446)	−1.61 (0.110)
Gender × MPH treatment	−2.30 (0.022)*	−1.39 (0.166)	0.51 (0.611)	−0.76 (0.447)	−2.41 (0.017)*	−0.04 (0.971)
ADHD subtypes × MPH treatment	0.91 (0.363)	−0.14 (0.886)	−0.33 (0.741)	−0.07 (0.943)	−0.01 (0.990)	−0.99 (0.322)

Note: Data are expressed as statistical values, t (p-value), using linear mixed model analysis; ^a^ ADHD subtypes: inattentive type vs. hyperactive-impulsive type and combined type; *p<0.05, **p<0.01.

## Discussion

This study demonstrates that commission errors and d’ significantly improved during MPH treatment, while the other indices of TOVA did not. This finding suggests that ADHD patients have an improved ability of impulse control and perceptual sensitivity, but not sustained attention or response speed, under long-term MPH treatment. Our previous study has established that one dose of MPH produced more effects on impulsivity than on inattention in children with ADHD [13]. A previous study involving the long-term effects of MPH on TOVA performance, in which participants were also tested off the drug, also demonstrated a compatible finding [16]. Similarly, a 6-month open-label study showed that children with ADHD receiving MPH improved more in impulsivity dimensions than in attention dimensions of the Conners’ Continuous Performance Test, which were tested while on the medication [23]. Moreover, another study found that neurocognitive effects of MPH were more prominent on tasks without an executive function component than with an executive function component [8]. In summary, the stimulant-related cognitive enhancements may be discrepant in different dimensions of neurocognitive function.

Results of this study also suggest that the effects of MPH on two TOVA indices can be sustained for at least 12 months, even when ADHD patients are tested at drug-free status. These results support the conclusion of a recent review that MPH is continuously effective for an extended period [10]. Some long-term follow-up studies for ADHD patients have found that treatment with medication in childhood may improve some measures of academic achievements [32,33]. Biederman *et al.* demonstrated that non-medicated ADHD patients have a more pervasive pattern of cognitive deficits than medicated ADHD ones [34]. In contrast to the studies in self-selected naturalistic settings, the Multimodal Treatment study of children with ADHD (MTA), which conducted with a randomized-controlled design, reported contradictory findings. Although the benefits of stimulant medications persisted for 14 months [35], the earlier advantage of having continuous intake of medication for 14 months is no longer apparent at the 36-month follow-up [36]. Therefore, the likelihood that selection bias inflated the long-term neurocognitive effects of MPH in previous studies can not be ruled out. Further research is warranted to clarify whether the MPH-related cognitive enhancements exist during long-term treatment, even when the drug effect diminishes.

This study also examines whether differences occur in neurocognitive performance between age, gender and ADHD subtypes during MPH treatment. Analytical results indicate that older patients (≥9 y/o) performed worse in many indices of TOVA than younger ones (<9 y/o), and the older patients more significantly improved in response time during treatment. However, there were differences in some indices of TOVA at baseline between age groups (data not shown). The effect of age in neurocognitive performance is not necessarily implying that older patients gained more from MPH treatment than younger ones, and might be just a regression to the mean. Moreover, male patients more significantly improved in omission error and d’ than female ones during MPH treatment. Despite similar findings found in a previous study on the acute effects of MPH on TOVA performance [13], a recent study found that gender groups did not differ in neuropsychological performance modulated by MPH [37]. The current studies regarding the interaction of age, gender and MPH treatment effects are still scarce. ADHD symptoms or sample selection may confound differences in neurocognitive effects between age or gender groups. Further study is thus warranted on whether age and gender serve as predictors of MPH-related cognitive improvements.

Results of this study further demonstrate that patients with ADHD-inattentive type had a higher ADHD composite score in TOVA than patients with hyperactive-impulsive or combined type. Meanwhile, MPH treatment and ADHD subtypes did not interact with each other in all TOVA indices. Subjects with hyperactive or combined type of ADHD show more significantly improve in commission errors than those with inattentive ADHD after one dose of MPH [13]. However, several longitudinal studies found that ADHD subtypes do not differ in MPH enhanced cognitive performance [21-23]. We can infer that the diversity of results from studies of MPH effects on the neuropsychological profiles of subtypes of ADHD patients may reflect variations in methodology. This study does not support discriminated effectiveness in terms of neurocognitive performance between ADHD subtypes.

Despite its contributions, this study has certain limitations. First, a comparison group was absent. Exactly how age-related decline in ADHD-symptoms and social and environmental influence affects TOVA performance could thus not be controlled. Longitudinal studies have shown that there is a natural decline of symptoms with age, particularly the symptom of hyperactivity and impulsivity, in children with ADHD [38]. Therefore, the improvements in commission errors and d’ observed in our study might be accounted for an age-related maturation. Second, the subjects performed TOVA 3 times, with 6-month time intervals between each TOVA tests. Practice effects may be minimal, yet could not be completely ruled out. Third, the treatment procedure of MPH was not strictly standardized, and drug adherence was not measured systematically. Furthermore, a high rate of early termination, variation of MPH dosage and possible inadequate drug adherence may influence the estimation for the extent to which MPH affects TOVA performance.

## Conclusions

Using TOVA as a neuropsychological assessment scheme revealed that impulsivity and perceptual sensitivity in ADHD patients significantly improved during MPH treatment for 12 months. Age and gender, yet not ADHD subtypes, appear to influence the MPH treatment effects in some indices of TOVA. A future study containing a comparison group is suggested to confirm whether the neurocognitive improvements are attributed to long-term effects of MPH or natural maturation of patients.

## Competing interests

The authors report no competing of interest.

## Authors’ contributions

YSH and LJW are joint first authors and contribute equally to this manuscript. YSH conceived the study and recruited the participants. LJW analyzed the data and wrote the paper. CKC carried out the literature search and helped to draft the manuscript. All authors read and approved the final manuscript.

## Pre-publication history

The pre-publication history for this paper can be accessed here:

http://www.biomedcentral.com/1471-244X/12/194/prepub
